# Characterisation of the transient mechanical response and the electromyographical activation of lower leg muscles in whole body vibration training

**DOI:** 10.1038/s41598-022-10137-8

**Published:** 2022-04-14

**Authors:** Isotta Rigoni, Tecla Bonci, Paolo Bifulco, Antonio Fratini

**Affiliations:** 1 Biomedical Engineering, College of Engineering and Physical Sciences, Aston University, Birmingham, UK; 2 EEG and Epilepsy Unit, Clinical Neuroscience Department, University Hospital and Faculty of Medicine of Geneva, Geneva, Switzerland; 3 Department of Mechanical Engineering and the Insigneo Institute for in Silico Medicine, University of Sheffield, Sheffield, UK; 4 Department of Electrical Engineering and Information Technology, University of Naples Federico II, Via Claudio 21, 80125 Naples, Italy

**Keywords:** Biomedical engineering, Neurophysiology

## Abstract

The aim of this study is to characterise the transient mechanical response and the neuromuscular activation of lower limb muscles in subjects undergoing Whole Body Vibration (WBV) at different frequencies while holding two static postures, with focus on muscles involved in shaping postural responses. Twenty-five participants underwent WBV at 15, 20, 25 and 30 Hz while in hack squat or on fore feet. Surface electromyography and soft tissue accelerations were collected from Gastrocnemius Lateralis (GL), Soleus (SOL) and Tibialis Anterior (TA) muscles. Estimated displacement at muscle bellies revealed a pattern never highlighted before that differed across frequencies and postures (p < 0.001). After stimulation starts, muscle oscillation peaks, drops and further stabilises, suggesting the occurrence of a neuromuscular activation to reduce the vibration-induced oscillation. The oscillation attenuation at the SOL muscle correlated with its increased activation (rho = 0.29, p < 0.001). Furthermore, only specific WBV settings led to a significant increase in muscle contraction: WBV-induced activation of SOL and GL was maximal in fore-feet (p < 0.05) and in response to higher frequencies (30 Hz vs 15 Hz, p < 0.001). The analysis of the mechanical dynamics of lower leg muscles highlights a resonant response to WBVs, that for the SOL correlates to the increased muscle activation. Despite differing across frequencies and postures, this resonant behaviour seems to discourage the use of dynamic exercises on vibrating platforms. As for the most efficient WBV combination, calf muscle response to WBVs is maximised if those muscles are already pre-contracted and the stimulation frequencies are in the 25–30 Hz range.

## Introduction

Whole Body Vibration (WBV) refers to the use of mechanical stimulation, in the form of vibratory oscillations extended to the whole body, to elicit neuromuscular responses in multiple muscle groups^1^. This approach has become increasingly popular as it evokes large muscle responses and, more importantly, it elicits muscle activity through physiological pathways, improving the overall motor performance while enhancing strength and flexibility^2–7^.

Although the effective mechanisms behind neuromuscular outcomes in WBV have been debated for long time^1,2,8^, those that go under the names of tonic vibration reflex (TVR) and muscle tuning are the most accredited for.

It is well known that, when vibrations are applied directly to tendons or muscle bellies (i.e., focal vibrations), fibres length changes activating a reflex increased motor-unit (MU) firing rates^9,10^, phased-locked specifically to the vibratory cycle—namely TVR^9,11,12^. WBV stimulation is characterised by a much lower frequency range (15 to 45 Hz) than typical focal stimulations (> 100 Hz) and vibrations are generally delivered to the lower limbs via the use of platforms on which subjects stand: being a mechanical stimulus, vibrations are transferred through the kinematic chain determined by the body posture^13–15^. To confirm the occurrence of the TVR mechanism during WBV stimulation, different studies compared muscle latencies during WBVs and during manoeuvres that knowingly trigger TVR, but yielded conflicting results^13,16,17^, leaving the quest unresolved.

Previous findings suggest that, when exposed to vibrations, muscles contract to reduce the soft-tissue resonance, especially when the stimulation frequency, *ω*_*a*_, is close to their natural one^18–20^. This process is known as muscle tuning and is perpetrated by muscles to minimize the soft-tissue vibrations that arise when walking or running^21,22^. Since the same tuning behaviour was observed in response to pulsed and continuous vibrations^19^, it has been proposed as one of the possible body reactions to WBV^23,24^.

The natural frequency of a system depends on its mass, $m$, and stiffness, $k$ according to the formula *ω*_*0*_=$\sqrt{\mathrm{k}/{\mathrm{m}}^{2}}$. While the mass of a muscle is constant, its stiffness can be modulated by both muscle activation and the specific body posture. Changes in subject posture do therefore change muscle stiffness, leading to a change in their natural frequency, and this represents the main drawback of dynamic exercises on a vibrating platform^23^. The body kinematic chain involved in the transmission of the vibrations changes continuously, impeding a consistent stimulus delivered at the specific muscle group. During static WBV exercises instead, the kinematic chain remains constant and the tuned muscle contraction is one of the main mechanisms left to dampen vibrations.

The use of static versus dynamic exercises is only one of the main variables that can be changed across studies and that accounts for the huge variability of results reported. Indeed, although promising results of WBV training are reported in the literature^25–33^, the systematic use of such approach in training and rehabilitation practices is jeopardized by few discording findings^34–36^. Conflicting results are likely to be related to the high amount of variability in WBV settings used throughout different studies, while still lacking of standardised training protocols. Frequency, acceleration, amplitude and duration of the stimulus play a fundamental role in WBV stimulation as well as subject posture—a key variable to be considered in understanding transmissibility of the stimulus^37–39^. Moreover, most of the WBV literature generally misses the mechanical dynamics analysis at the target muscle, failing to characterize the relationship between muscle oscillation and activation, or focuses on local oscillations and muscular activity when the dampening has probably already happened^40^, which may hide important information about the dynamics of the underlying systems after the vibration onset.

With this study we aimed at characterising, since stimulus onset, the transient mechanical response of lower limb muscles in subjects undergoing WBV stimulation. We also investigated the neuromuscular activation of such muscles: we focussed our attention on calf muscles—soleus (SOL), gastrocnemius lateralis (GL) and tibialis anterior (TA)—for their relevance to postural control training.

We hypothesised that muscles would require an intrinsic time interval to react to the vibratory stimulation and tune muscle stiffness accordingly, based on a given stimulus and body posture. In addition, we hypothesised that the extent of vibration dampening is related to the increase of muscle activity. To test our hypothesis, we recorded and analysed muscle displacement—derived from accelerometers placed on muscle bellies—and muscle activation in response to WBVs delivered via a side alternating platform at different frequencies while the subjects held two static postures usually reported in WBV training.

## Materials and methods

### Participants and experimental design

Seventeen females and eight males (age 24.8 ± 3.4 years; height 172.0 ± 8.6 cm; mass 64.6 ± 10.5 kg) volunteered in the study after providing written informed consent. History of neuromuscular or balance disorders as well as recent injuries were among the exclusion criteria. To evaluate muscle activation and displacement during WBV, surface electromyography (sEMG) signals and accelerations were collected from three lower limb muscles during two static exercises performed in static conditions (without WBV—hereafter called baseline activity) and when different vibration frequencies were delivered. The protocol of the study received approval by the Ethics Committee of the School of Life and Health Sciences at Aston University (reference number: 1439).

Pairs of Ag/AgCl surface electrodes (Arbo Solid Gel, KendallTM, CovidienTM diameter of 24 mm, centre-to-centre distance 24 mm) were placed over the Gastrocnemius Lateralis (GL), Tibialis Anterior (TA) and Soleus (SOL) muscles of the dominant leg, as suggested in the SENIAM guidelines^41^. The reference electrode was placed on the styloid process of the right ulna. The EMG data were sampled at 1000 Hz (PocketEMG, BTS Bioengineering, Milano, Italy) and sent wirelessly to a laptop via the Myolab software, version 2.12.129.0 (BTS Bioengineering, Milano, Italy).

Accelerations were measured via tri-axial accelerometers (AX3, Axivity Ltd, Newcastle, United Kingdom; range =  ± 16 g, sampling frequency = 1600 Hz) placed on GL, TA and SOL muscle bellies, next to the EMG electrodes. The accelerometers were aligned with the x-axis parallel to the longitudinal axis of the leg segment, the z-axis normal to the skin surface and the y-axis perpendicular to the x–y plane. Accelerations were recorded using the dedicated open source software OMGUI developed by Newcastle University^42^.

### Whole body vibration stimulation protocol

Subjects underwent the WBVs barefoot. The WBVs were delivered via a side-alternating platform (Galileo^®^ Med, Novotec GmbH, Pforzheim, Germany), as it was shown to evoke bigger neuromuscular activations than synchronous vibrating ones^37^: a peak-to-peak amplitude of 4 mm was used. For each subject, ten trials were collected to evaluate the effect of five stimulation frequencies that covered the frequency range offered by the platform—0, 15, 20, 25, 30 Hz—and two subject postures: hack squat (HS) and fore-foot (FF). To ensure heels off the ground during the FF trials, subjects were asked to keep their heels in contact with a parallelepiped-shaped foam (30 × 4 × 3 cm) glued on the platform while keeping their lower limb straight. During HS trials instead, subjects were asked to keep their knees flexed at about 110° and a goniometer was used to check the angle at the beginning of each HS trial. Trials were administered in a random order with a one-minute break between consecutive trials.

Hereafter, trials with vibratory stimulation are referred to as the “WBV trials” $({HS}_{15}$ ${HS}_{20}$ ${HS}_{25}$ ${HS}_{30}$ ${FF}_{15} {FF}_{20}$ ${FF}_{25}$ ${FF}_{30}$) and the others as the “baseline trials” (${HS}_{0}$ and ${FF}_{0}$). WBV trials consisted of 40 s: recordings contained 10 s with no vibration (${WBV}_{off}$ portion), once the subject acquired the prescribed posture, followed by 30 s of WBVs at the prescribed frequency (${WBV}_{on}$ portion). Baseline trials were used to assess the relevant subject-specific EMG baseline activity (${HS}_{0}$ and ${FF}_{0}$) over a 30 s period.

Twelve Vicon Vero v2.2 optical cameras (Vicon Nexus, Vicon Motion Systems Limited, Oxford, UK) were used to measure subjects’ posture and assure consistency throughout the experiment. Sixteen retroreflective markers were attached to the participant’s body, according to the Plug-In-Gait Lower-Limb model^43^. Data were sampled at 100 Hz and knee and ankle angles were obtained by extracting the kinematics in the sagittal plane using the proprietary software. Specifically, the ankle and knee angles were used to check for consistency across conditions and subjects.

### Data processing and features extraction-acceleration data

Raw accelerations from WBV trials were analysed in Matlab^®^R2019a (The Mathworks, Inc., Natick, MA). Accelerations were band-pass filtered between 10 and 100 Hz to remove gravity components and accommodation movements, usually confined between 0 and 5 Hz^44,45^, and to retain only vibration-induced muscle displacements, located mostly at the stimulation frequency and its superior harmonics^46^. Filtered epochs were then double integrated to estimate local displacement (Eq. 1) along the different axes (${disp}_{x}, {disp}_{y}, {disp}_{z}$) and the total displacement recorded at each muscle level was estimated as:

1 $${DISP}_{TOT}\left(\mathrm{t}\right)=\sqrt{{{disp}_{x}(t)}^{2}+{{disp}_{y}(t)}^{2}+{{disp}_{z}(t)}^{2}},$$

where $t=\mathrm{1,2},\dots ,N$, with $N$ being the total number of samples.

**Figure 1:**
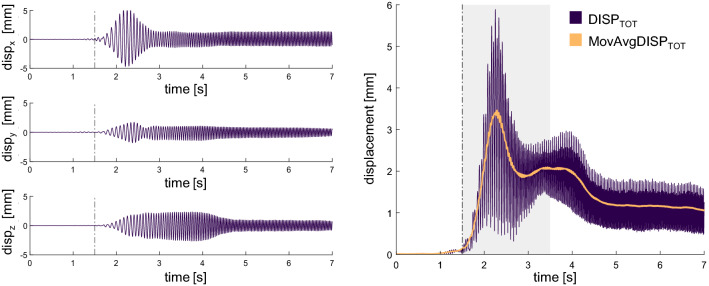
Extraction of muscle dynamics. On the left panel, muscle displacement obtained from double integration of the soft-tissue acceleration recorded at the GL site, ${\mathrm{HS}}_{30}$. Displacement along time is reported for the x, y and z axis. On the right panel, the GL total displacement obtained from the combination of the signals on the left (in purple): the moving average is depicted in orange. The vibration onset is indicated on the graphs by the vertical dashed line; the two-second interval used for the search of ${\mathrm{t}}_{\mathrm{P}}$ is highlighted with a grey area. The red and green asterisk indicate ${\mathrm{t}}_{\mathrm{P}}$ and ${\mathrm{t}}_{\mathrm{A}}$ respectively.

**Figure 2:**
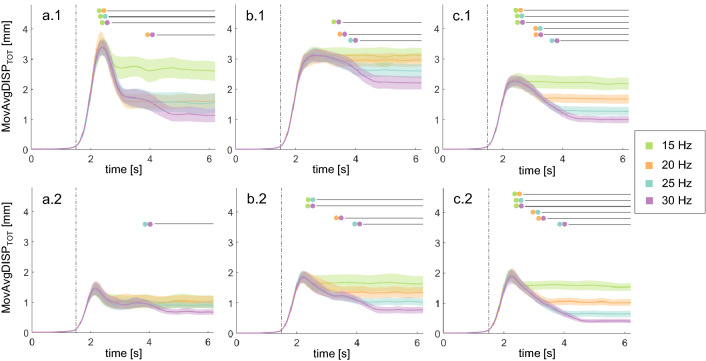
Muscle dynamics during WBVs at different frequencies and postures. Moving average of the total displacement (mean ± standard error) for each muscle (a = GL, b = SOL, c = TA) (N = 25). The top row (.1) shows the mechanical responses while subjects underwent the WBVs in Hack Squat; the bottom row (.2) shows the responses while subjects were in Fore Feet. The results of the cluster-based permutation tests are indicated by the black lines (p < 0.003125) and the conditions considered for each comparison are listed via the colour-wise legend. The vertical dotted line represents the vibration onset. The orange (20 Hz) and blue (25 Hz) signals overlap in a.1.

To track the low-frequency mechanical muscle response to WBVs, a moving average of ${DISP}_{TOT}$ (${MovAvgDISP}_{TOT}$) was calculated using a 250 ms sliding window (Fig. 1). To compare and superimpose muscle displacement among different subjects, ${MovAvgDISP}_{TOT}$ vectors were time-locked to the point where a 0.1 change in the slope was detected, which will be hereafter referred to as the vibration onset, and used for statistical analyses. To describe muscle response to vibrations, two time points were defined as follows:

- ${t}_{P}$: time-point in correspondence of the peak, defined as the maximum value of each ${MovAvgDISP}_{TOT}$ signal in a 2-s interval after the vibration onset (grey area in Fig. 2);
- ${t}_{A}:$ common attenuation time-point among the different muscles (Eq. 2), chosen to represent the response following the peak phase, where muscle displacement is stabilised and minimised, reaching a steady-state. The time-point representing the ending of the peak (${t}_{EndPeak}$) was first computed for each muscle and trial: ${MovAvgDISP}_{TOT}$ signals were averaged across subjects and low-pass filtered at 10 Hz. A 0.5 s sliding window search was applied to identify the slope change time point (${t}_{EndPeak}$), with a threshold of 0.06. Once a ${t}_{EndPeak}$ was identified for all muscles and conditions, ${t}_{A}$ was defined as:
  2 $${t}_{A}={round(max(t}_{EndPeak})+{\frac{1}{2}\times max(t}_{EndPeak})),$$
  where ${max(t}_{EndPeak})$ is the longest peak duration observed across muscles and conditions. Since the longest peak duration was of 3.12 s, which was recorded for the GL in ${HS}_{30}$, ${t}_{A}$ was located 4.7 s after the vibration onset (green asterisk in Fig. 1).

To quantify the extent of the displacement attenuation at each muscle site, ${ATT}_{DISP}$ (Eq. 3) was calculated for each subject as the difference between the maximum displacement recorded at that site and the steady-state one:

3 $${ATT}_{DISP}={MovAvgDISP}_{TOT}\left({t}_{P}\right)-{MovAvgDISP}_{TOT}\left({t}_{A}\right).$$

### Data processing and features extraction-EMG Data

To isolate the muscle activity preceding the stimulation (${WBV}_{off}$) from the one actually induced by the vibrations (${WBV}_{on}$), each WBV trial was split into two epochs: 10 and 30 s, respectively. The central portions of these signals (6 and 20 s, respectively) were extracted and retained for analyses. Similarly, the central 20 s of the baseline trials (${HS}_{0}$ and ${FF}_{0}$) were extracted and retained for analyses.

All epochs were band-pass filtered between 5 and 450 Hz with a 5th order Butterworth filter and a mean running root mean square ($rRMS$) value was obtained from both the baseline ($RM{S}_{baseline}$) and the ${WBV}_{off}$ epochs (${RMS}_{WBVoff}$).

To remove motion artefacts from ${WBV}_{off}$ and ${WBV}_{on}$ epochs^46^, a type II Chebyshev band-stop filter was applied at each stimulation frequency and its harmonics up to 450 Hz on the EMG spectra. This resulted in 30, 22, 18 ad 15 stop-band filters (Eq. 4) applied to epochs derived from WBV trials delivered at 15, 20, 25 and 30 Hz, respectively, following the calculation:

4 $$\#\mathrm{filters}=round\left(\frac{frequency \; spectrum \; upper \; limit}{stimulation \; frequency}\right).$$

For each WBV trial and muscle, two $rRMS$ vectors were computed on both artefact-free epochs (${WBV}_{off}$ and ${WBV}_{on}$)^46^ and used to calculate the relevant mean RMS values: ${RMS}_{WBVoff\sim }$ and ${RMS}_{WBVon},$ respectively. To compare the values obtained during the different trials, a factor (Eq. 5) taking into account the proportion of power removed by the comb-notch filter was calculated^47^:

5 $$Bias=\frac{{RMS}_{WBVoff\sim }}{{RMS}_{WBVoff}}$$

and was used to adjust (Eq. 6) ${RMS}_{WBVon}$ values:

6 $$adjRM{S}_{WBV}={RMS}_{WBVon}\times \frac{1}{Bias}.$$

To evaluate the WBV-induced increment of muscular activation, $RM{S}_{baseline}$ were subtracted from the $adjRM{S}_{WBV}$ obtained for the WBV trials in the respective posture (Eq. 7). These resulting values will be hereafter referred to as the $incrementRM{S}_{WBV}$:

7 $$incrementRM{S}_{WBV}=adjRM{S}_{WBV}-RM{S}_{baseline}.$$

In total, eight values were retained for each subject and used for statistical analysis.

### Statistical analyses

For each muscle, a cluster-based permutation test was used to compare the mechanical response of muscles over time^48,49^ for:

- ${MovAvgDISP}_{TOT}$ between the two postures at the four frequencies (${HS}_{15}-{FF}_{15}$; ${HS}_{20}- {FF}_{20}$; ${HS}_{25}- {FF}_{25}$; ${HS}_{30}- {FF}_{30}$)
- ${MovAvgDISP}_{TOT}$ between frequency pairs in HS and FF (${HS}_{15}-{HS}_{20}$; ${HS}_{15}- {HS}_{25}$; ${HS}_{15}- {HS}_{30}$; ${HS}_{20}- {HS}_{25}$; ${HS}_{20}-{HS}_{30}$; ${HS}_{25}- {HS}_{30}$, and in FF, for a total of twelve tests per muscle).

Time series comparisons were performed over the portion of the signals between the vibration onset and ${t}_{A}$ to include both the peak and stabilization phase and because no effect was expected before the WBVs. 5000 permutations were used to build the random distribution against which the test statistic of the actual signal were compared. An alpha level of 0.05 was used to identify the significant clusters for each comparison^50^. To overcome the multiple comparison problem introduced by the number of comparisons run for each muscle (4 + 12), the cluster p values were adjusted with a Bonferroni correction (p = 0.003125).

For each muscle, to test whether the electromyography activity increased significantly during the different WBVs, eight Wilcoxon signed rank tests (frequency (4) × posture (2)) compared the $incrementRM{S}_{WBV}$ to a normal distribution with zero mean and unknown variance. Analysis were performed in Matlab^®^R2019a.

For each analysed muscle, a two-way repeated measures Analysis of Variance (ANOVA) was conducted to examine the effect of the stimulation frequencies and subject postures on $incrementRM{S}_{WBV}$ [frequency (4) × posture (2)]. Bonferroni corrections were adopted for multiple comparisons. Since muscle responses were investigated per se, outliers were removed from the dataset of the specific muscle after visual inspection of the data. Residuals were inspected and the approximate normal distribution of the data was confirmed by the Anderson–Darling test^51^. Mauchly’s test of sphericity was used to assess the sphericity of the data: when the latter was not met, a Greenhouse–Geisser correction was applied. Analysis were run in SPSS 23.0 (IBM Corp., Armonk, NY, USA)^52^.

To relate the mechanical response with the physiological one, a Pearson correlation coefficient was calculated between ${ATT}_{DISP}$ and the outlier-free $incrementRM{S}_{WBV}$ population, after the subjects that were identified as outliers for ANOVA analyses were removed from the respective ${ATT}_{DISP}$ population. For each muscle, the data recorded in the eight trials (${HS}_{15}$ ${HS}_{20}$ ${HS}_{25}$ ${HS}_{30}$ ${FF}_{15} {FF}_{20}$ ${FF}_{25}$ ${FF}_{30}$) were pooled together.

### Ethics approval

The study was carried out according to the Declaration of Helsinki (2013) and was approved by the University Research Ethics Committee at Aston University (reference number: 1439).

### Consent to participate

All participants provided informed consent before participating.

### Consent for publication

All co-authors were aware of the publication of this study.

## Results

All subjects were compliant with the stimulation protocol: no adverse effects, such as fatigue or dizziness, were reported. The average ankle angles measured in FF were − 9.4° ± 6.4° where a negative measure indicates a plantar flexion. When participants underwent the WBVs in HS, the average knee angle was 70.8° ± 4.4°.

Muscles dynamics differed significantly depending on the posture and frequency: overall, a larger displacement was observed in HS trials and at lower frequencies.

A characteristic mechanical peak is visible shortly after the start of the stimulations in both postures, with the only exceptions of TA and SOL muscles when stimulated at 15 Hz. Peaks varied among muscles, postures and frequencies, but the displacement showed a similar trend, identified for this first time in this work: a peak with a successive drop and a further stabilisation after some seconds (Fig. 2).

GL attenuation after the peak was found to be significantly bigger at higher frequencies—at 20, 25 and 30 Hz rather than at 15 (p = 0.0002) and at 30 Hz rather than at 20 (p = 0.0014) (Fig. 2a.1) in HS. In FF, a similar trend was recorded: a greater attenuation was found at 30 Hz with respect to 25 Hz (p = 0.0004) (Fig. 2a.2).

Also the attenuation recorded at the SOL site was higher at 30 Hz than at 15 (p = 0.0006), 20 (p = 0.0002) and 25 Hz (p = 0.0014) while in HS (Fig. 2b.1). Similarly, in FF, a larger dampening was recorded at 30 Hz than at 15 (p = 0.0002), 20 (p = 0.001) and 25 Hz (p = 0.0002) and at 25 Hz than at 15 Hz (p = 0.0006) (Fig. 2b.2).

The mechanical response of TA confirmed the trend observed for the other muscles: its displacement attenuation after the peak was always higher at higher frequencies in HS (greater attenuation at 20 Hz than at 15, p = 0.0004; at 30 Hz than at 25, p = 0.0006; for all other comparisons, p = 0.0002) (Fig. 2c.1) and in FF (p = 0.0002) (Fig. 2c.2).

**Figure 3:**
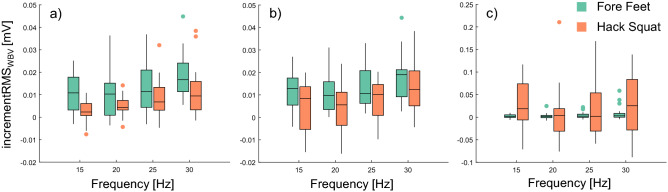
sEMG RMS ANOVA results. Box plots of incrementRMS_WBV_ values at different stimulation frequencies (15–30 Hz) of a = Gastrocnemius Lateralis (N = 21), b = Soleus (N = 21), and c = Tibialis Anterior (N = 22) are shown. Different colours are used to distinguish between the muscle responses in hack squat (orange) and in fore feet (dark green) while the dots represent the outliers retained for the specific population. No significant interactions resulted from the ANOVAs. For significant main effects of stimulation frequency and subject posture refer back to the text. The figure was produced with Gramm^53^.

Normality of the $incrementRM{S}_{WBV}$ was confirmed in most of the conditions, for all three muscles. It was not always normally distributed for TA, but the latter distributions were similarly skewed to those that met normality. Four subjects were removed from the $incrementRM{S}_{WBV}$ dataset of GL and SOL, and three from that of TA, since represented outlier values. Distribution of $incrementRM{S}_{WBV}$ values for the different muscles, posture and frequencies is depicted in Fig. 3.

The Wilcoxon tests confirmed a significant WBV-induced muscle activation ($incrementRM{S}_{WBV}$) in all conditions for the GL (see first row of Table 1) and in most of the conditions for the SOL, apart from ${HS}_{15}$ (see second row of Table 1). Instead, the TA showed a significant response to WBVs only for 15 and 30 Hz and ${FF}_{25}$ (see third row of Table 1).

**Table 1:** Results of the Wilcoxon signed rank tests used to test whether the WBV-induced increment of muscle activation (${\mathrm{incrementRMS}}_{\mathrm{WBV}}$) was significantly different from zero in each condition. The mean (SD) of ${\mathrm{incrementRMS}}_{\mathrm{WBV}}$ measured in each condition is reported, as well as the p-value of each test (N = 25). The asterisk denotes statistical significance.

	HS				FF			
	15 Hz	20 Hz	25 Hz	30 Hz	15 Hz	20 Hz	25 Hz	30 Hz
GL	0.0026 (0.0067) p = 0.023*	0.0043 (0.0063) p = 0.002*	0.0091 (0.0077) p < 0.0001*	0.0160 (0.0154) p < 0.0001*	0.0158 (0.0269) p < 0.0001*	0.0120 (0.0139) p < 0.0001*	0.0193 (0.0298) p < 0.0001*	0.0329 (0.0744) p < 0.0001*
SOL	0.0031 (0.0157) p = 0.051	0.0031 (0.0143) p = 0.039*	0.0092 (0.0186) p = 0.002*	0.0179 (0.0157) p < 0.0001*	0.0203 (0.0372) p < 0.0001*	0.0159 (0.0171) p < 0.0001*	0.0194 (0.0217) p < 0.0001*	0.0325 (0.0641) p < 0.0001*
TA	0.0349 (0.0571) p = 0.012*	0.0128 (0.0622) p = 0.396	0.0229 (0.0633) p = 0.241	0.0310 (0.0644) p = 0.045*	0.0026 (0.0053) p = 0.021*	0.0023 (0.0080) p = 0.165	0.0044 (0.0090) p = 0.019*	0.0089 (0.0166) p = 0.01*

ANOVA analyses showed that although no significant interaction was found for the GL (N = 21), main effect of stimulation frequency (F(3, 60) = 14.397, p < 0.0001) and subject posture were statistically significant (F(1, 20) = 15.433, p = 0.001). Specifically, GL-sEMG activity increased more in FF than in HS (p = 0.001) and 30 Hz was the stimulation frequency that evoked the highest muscular activation when compared to 15 Hz (p < 0.0001) and 20 Hz (p = 0.001). The WBV-induced increment of GL activation was also higher at 25 Hz than at 20 Hz (*p* = 0.02). Similarly, no significant interaction was found for the SOL (N = 21) and a similar stimulation frequency (*F*(1.772, 35.434) = 12.982, p < 0.0001)) and subject posture (*F*(1, 20) = 6.357, p = 0.02)) main effects were found. The WBV-induced increment of SOL activity was higher in FF than in HS (p = 0.02) and 30 Hz was the stimulation frequency in which the highest sEMG increment was found when compared to 15 Hz (p = 0.002), 20 Hz (p = 0.004) and 25 Hz (p = 0.037). Moreover, a 25 Hz stimulation led to a higher muscle activation than 20 Hz (p = 0.009). No significant interaction nor main effect was instead found for the TA (N = 22).

**Figure 4:**
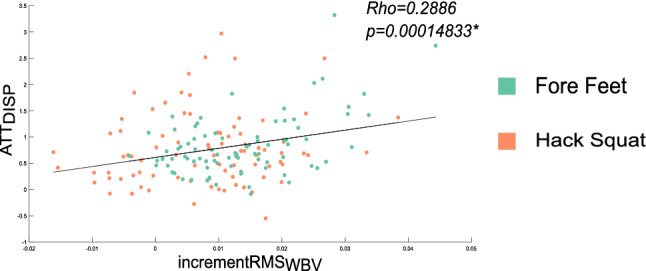
Correlation between SOL activity and displacement attenuation. Results of the Person correlation analyses performed between the increase of activation of SOL (incrementRMSWBV) and its respective displacement attenuation (N = 168). The asterisk depicts statistical significance.

A positive correlation was found between the increase of SOL muscle activity and the amount of displacement attenuation (rho = 0.2886, p < 0.001, see Fig. 4). No significant correlations were found between the augmented activation of GL and TA and the extent of displacement reduction measured at the respective site.

## Discussions

WBV stimulation has been studied for decades, yet a conclusive approach to adapt treatment parameters to the target muscles is missing. There is a plethora of studies dedicated to deciphering the relation between frequency, amplitude, acceleration and duration of the stimulus with the corresponding muscular outcomes.

Some of them relate the acceleration of the platform to muscle activity^54,55^, some others investigate its relation to body joint acceleration^39,56,57^, others more simply address the transmissibility of vibrations to the shank soft-tissue compartments^58,59^, only few reflect on the relation of the latter and muscle activity^60^, while no study was found to link the vibration at the muscle site to its own activation. Moreover, muscular and mechanical outcomes are generally assessed seconds after the vibration onset, failing to characterise the initial dynamic response to WBVs. To the authors knowledge, this is the first study to analyse the progressive dynamics of the displacement at the muscle site and EMG activation while undergoing WBVs with different static postures.

Our analysis of the dynamics of the displacement and EMG recorded at each muscle site confirmed our hypothesis: muscle reaction to WBVs depends on stimulus characteristics and subject’s posture and develops in time to reduce muscle oscillations. Indeed, a common mechanical pattern, never highlighted before, can be observed from our results (Fig. 2). In response to vibratory stimulation, the extent of oscillations of muscles shows a rising phase, a peak and a subsequent drop, all of which completed within 4 to 5 s after the vibration onset, followed by a sustained stable oscillation (plateau). Neither the stimulation amplitude nor the posture of participants varied during individual tests, hence a neuromuscular response is accounted for the observed dynamics. This interpretation aligns to the muscle tuning theory, whereby soft-tissue oscillations arising in response of impact forces applied to the feet are dampened by an increase in muscle activation^20,21,61^. During WBVs, in fact, vibrations are transferred from the feet to the muscles via the body kinematic chain and produce soft-tissue compartment oscillations at the stimulation frequency, which in our case was in the range of the natural frequencies of calf muscles^18^. In light of the reported theory, it is therefore reasonable to assume that, if a resulting potential resonance is detected, muscle contraction is increased to avoid damage, creating the characteristic raising and falling curves observed in our recordings.

The differences observable in these curves confirm that mechanical response changes across muscles, frequencies and posture, suggesting that it is not of artefactual nature but that it actually reflects an underlying muscular activation. Moreover, they also suggest that not all combinations of frequencies and postures can elicit a significant resonant response in some muscles. This resonant response in fact absent in the SOL muscle at 15 Hz (in both FF and HS) and at 20 Hz in HS. Similarly, no peak is observable in the TA muscle at 15 Hz WBVs delivered in HS. These results resemble those obtained by Pollock et al. (2010), where 15 Hz represented the upper limit for transmissibility of vibrations from the platform. The accelerations at the knee joint were found to peak at 15 Hz and to dramatically decrease with increasing frequencies, suggesting the occurrence of muscle tuning^39^. Similarly, vibration transmission to the triceps surae and thigh muscle compartments were found to consistently decrease with increasing frequencies^58^, suggesting that a damping effect was more present at frequencies that are closer to the muscles’ resonant ones (the higher ones). These results are in line with what we observe in the plateau phase of the mechanical response, nevertheless, these conclusions were drawn on partial information analyses (the central interval of the WBV trials), and do not include the analysis of the initial dynamics.

Our study advances the understanding of muscles reaction to WBVs according to stimulation characteristics and, specifically, highlights that only after an intrinsic interval, which in this study is around 5 s, this reaction can completely settle. This may also explain why static exercises (postures) are found to be more effective than the dynamic ones while on the vibrating platform^23^: during the first, muscles can tune to WBVs as opposed to a continuously changing kinematic chain, with changes of muscle contraction and sensitivity to vibrations^10^.

In addition, the analysis of the physiological response of muscles to WBVs highlighted specific combinations of posture/frequency able to produce maximal results. As expected from acceleration analyses, also muscles activation varied: GL sEMG activity was significantly enhanced in all WBV combinations, while only specific combinations were effective for SOL and TA activation. This confirms the importance of the selection of appropriate WBV parameters combinations to activate target muscles. In addition, undergoing WBV stimulation while in FF was found to lead to a higher increase of GL and SOL sEMG activity rather than in HS position, confirming previous research findings^37^. According to the TVR theory, contracted muscles are in fact more responsive to vibrations^10^, and in our case GL and SOL, both plantar-flexors, are more engaged in FF than HS^62,63^, suggesting that, as well as muscle tuning, the TVR might have played a role in shaping muscular responses to WBVs. WBVs delivered at 30 and 25 Hz triggered a greater activation in both muscles, as similar findings reported^64^, supporting the previous proposal of GL natural frequency residing between 25 and 30 Hz^65^. These conclusions are further confirmed by the observation of the permutation test results. Most differences were appreciable for the plateau phase, where the attenuation of GL and SOL soft-tissue compartment displacement was significantly higher at 30 Hz than at other WBV frequencies, further supporting the claim that this frequency is the one triggering the largest tuning effect. Moreover, the positive correlation between the SOL sEMG increase and the displacement attenuation further suggest that the reduction of displacement in the plateau phase is indeed the manifestation of a neuromuscular response, potentially activated to reduce resonance. The absence of correlation in GL might be explained by the limited range of frequencies investigated. Recent results in fact suggest that correlation between vibration transmissibility and activation of the gastrocnemius lateralis are significant when including frequencies up to 40 Hz^60^. Similarly, the absence of any posture or frequency effect on the TA activation during WBVs might be explained by the following: (i) the stimulation frequencies used in this study that were limited to 30 Hz and not enough close to TA’s natural frequency, which ranges up to 50 Hz^19^; (ii) the selected postures that did not lead to an appropriate level of TA engagement, limiting its response to WBVs^10^; (iii) the phasic nature of the TA, which makes it physiologically different from the other muscles included in this study^66^.

For further studies on the topic, synchronisation of EMG recordings, soft tissue and platform accelerations should be carefully considered and justified. Vibration propagation does in fact depends not only on the level of stiffness of muscles, but also on the gender and anthropometrics of the subjects^67^. With the procedure adopted in this study, it was possible to align the soft-tissue accelerations/EMG recordings at the time where the tissue begins to oscillate (rather than on the platform onset). This allowed a more appropriate synchronisation of muscle activity and mechanical response between subjects.

## Conclusions

Our results highlighted a muscle driven mechanical response in muscles undergoing vibratory stimulation: a clear peak followed, after few seconds from the stimulus onset, by a more stable plateau that reflects a “delayed” neuromuscular activation to modify the properties of the biomechanical system (e.g. muscle stiffness). Furthermore, EMG analyses highlight that calf muscles produce maximal response if participants are standing on the fore feet during stimulations in the range of 25–30 Hz (its natural frequencies). Our results also suggest that to elicit a stable muscle contraction while using a vibrating platform, training programmes should consider only static postures, or in alternative, participants should be instructed to hold the same posture for longer than five seconds. This approach therefore may have profound impact on training or rehabilitation protocols aiming towards postural and balance improvement or recovery.

## Data Availability

Since sharing data in an open-access repository was not included in our participant’s consent and therefore compromises our ethical standards, data are only available on request from the corresponding author.

## Code availability

The code used for the analyses of the data will be shared upon request to the corresponding author.
